# Editorial: Role of Endophytes in Plant Health and Defense Against Pathogens

**DOI:** 10.3389/fpls.2020.01312

**Published:** 2020-08-26

**Authors:** Massimiliano Morelli, Ofir Bahar, Kalliope K. Papadopoulou, Donald L. Hopkins, Aleksa Obradović

**Affiliations:** ^1^ Consiglio Nazionale delle Ricerche, Istituto per la Protezione Sostenibile delle Piante, Sede Secondaria di Bari, Bari, Italy; ^2^ Department of Plant Pathology and Weed Research, Agricultural Research Organization (ARO), Rishon LeZion, Israel; ^3^ Department of Biochemistry and Biotechnology, University of Thessaly, Larissa, Greece; ^4^ University of Florida, Gainesville, FL, United States; ^5^ Faculty of Agriculture, University of Belgrade, Belgrade, Serbia

**Keywords:** endophytes, metagenomics, plant defense response, bioactive compounds, bioinoculants, plant growth-promoting bacteria

## Introduction

With the increasing social concern in avoiding, or at least reducing, the application of pesticides and chemical fertilizers, in favor of sustainable eco-friendly alternatives, the search for beneficial microorganisms and microbial-derived compounds has become one of the most popular Research Topics in the field of plant-microbe interactions (Cardoso Filho, 2019; Omomowo and Babalola, 2019).

Bacterial and fungal endophytes ubiquitously inhabit plant tissues without causing any adverse effect. On the contrary, their presence is often of benefit for the host, as they improve tolerance to abiotic adversities, enhance growth, and, relevantly, can modulate plant immune response and suppress pathogen colonization (Dini-Andreote, 2020). Since endophytic microorganisms typically cover the same ecological niches occupied by fungal and bacterial phytopathogens, they have been widely proposed as biocontrol agents that could be used as an alternative to pesticides (Compant et al., 2013).

Thanks to the multifaceted role they play, endophytic microbial resources are now considered crucial in the perspective of their potential use to achieve sustainable improvements in the agro-food system. As a consequence, there is now a scientific ferment trying to analyze every aspect of their interaction with plants and associated pathogens.

With 16 Original Research Articles and one Review, this Research Topic provides an overview of the current state of the art on the large research effort currently dedicated to understanding the role of endophytes in plant health and defense against pathogens.

## A Crosstalk With Plant Defense Pathways

Among the most challenging aspects resulting from the investigation on the application of endophytes, and in particular of plant growth-promoting bacteria (PGPB), there is the ability of several strains to trigger plant defense mechanisms (Ma, 2017). Commonly, PGPB-induced systemic resistance (ISR) is found to be associated with the up-regulation of genes involved in the pathways of jasmonic acid and ethylene (Pangesti et al., 2016). Biochemical responses such as increased synthesis of reactive oxygen species (ROS) and phenolic metabolites (Benhamou, 1996; Samain et al., 2017) are often associated with ISR, as well as anatomical modifications like the deposition of callose and lignin in the endophyte-colonized tissues (Benhamou, 1996; Constantin et al., 2019).

The blurred distinction between ISR and the pathogen-induced systemic acquired resistance (SAR) (Van Loon et al., 1998) was manifested in the study of Samain et al., in which certain *Paenibacillus* strains (i.e. PB2), when used to control *Mycosphaerella graminicola*, induced up-regulation of genes, such as pathogenesis-related proteins (PR1) and chitinases, usually considered as markers of SAR. This peculiar induced resistance is of interest, as it may be a more usual phenomenon, previously observed in other PGPB genera, like *Bacillus* and *Pseudomonas* (Park and Kloepper, 2000; Trotel-Aziz et al., 2008; Samain et al., 2017) and confers wheat a durable resistance. Its mode of action, which appears to be significantly influenced by the pathogen strain, the plant growth phase, and its genotype, needs to be addressed in further detail.

The duration of the resistance effect to pathogen-induced biotic stresses that endophytes may activate is, indeed, a key point in management strategies. In highlighting the ability of *Rhizobium etli*, a common bean symbiont to activate robust defense responses against the pathogen *Pseudomonas syringae* pv. *phaseolicola*, Diaz-Valle et al. noted that *R. etli*-primed plants seem to develop a transgenerational defense memory. The persistence of this capability in F1 generation appears to be related to transcription factors, independent from ethylene signaling pathway, and again, involved in the activation of PR gene expression, as already proposed by (Huang et al., 2016).

Although in recent years associative symbioses have been widely studied in several beneficial bacteria (Ahemad and Kibret, 2014; Coutinho et al., 2015), relatively few studies analyzed their effects on the transcriptional response of plants. Relying on an established model of symbiosis, that constituted by rice and *Burkholderia sensu lato* (s.l.) (Cottyn et al., 2001; Mannaa et al., 2019), King et al. have described the differences in transcriptional regulations induced by two closely related PGPB with different phylogenetic and ecological backgrounds. Each strain induced a unique expression pattern in the jasmonic acid signaling pathway, and, interestingly, differences have been related to distinct colonization strategies.

Biochemical changes triggered in plants challenged with PGPB are often combined with anatomical alterations. Rodriguez et al. have shown that *Gluconacetobacter diazotrophicus* is capable of inducing a series of structural changes in inoculated *Arabidopsis thaliana* seedlings through the deposition of callose. As a result of this sclerosis in root, stem, and leaf tissues, the plant reinforces cell wall and withstands colonization by *Ralstonia solanacearum* responsible for wilt disease.

## Cooperative Endophyte-Mediated Resistance

The study by de Lamo and Takken focuses on an interesting example of endophyte-mediated resistance (EMR), a mechanism that seems distinct from ISR and SAR, as jasmonate, ethylene, and salicylic acid are not involved (Pieterse et al., 2014; Constantin et al., 2019). In this case, the target plant (tomato) established a tri-partite interaction with two different strains of the same root-invading fungus (*Fusarium oxysporum*). While pathogenic strains employ host-specific effectors to interfere with host immune signaling (van Dam et al., 2018), co-inoculation of pathogenic *Fusarium* strains with endophytic *Fusarium* strains induces resistance responses and reduces negative disease effects. This kind of tri-partite interactions that leads to EMR was also investigated by Del Barrio-Duque et al. In the attempt to identify bacterial strains that stimulate the growth of the beneficial fungus *Serendipita indica* (Varma et al., 1999; Gill et al., 2016), the authors found that strains belonging to *Mycolicibacterium* genus might boost the beneficial effects triggered by *S. indica* on tomato plants, while decreasing severity of the symptoms caused by *F. oxysporum* and *Rhizoctonia solani*. This example of cooperation in triggering the host response is intriguing, as it could be explained by the presence in the helper’s genome of several genes involved in vitamin and secondary metabolite production that might supplement *S. indica* bioenergetic capacity. Thus, cooperation among cross-talking microbial players may be needed to restrain pathogen colonization (Zuccaro et al., 2011; Salvioli et al., 2016).

## Endophytes Produce a Variety of Exploitable Bioactive Metabolites

An interesting facet of the interaction between endophytes and their hosts is the capacity of many microorganisms to improve the plant’s resistance by providing several bioactive metabolites (Gunatilaka, 2006). In some cases, the release of volatile compounds emitted by plant-associated bacteria has risen to the fore as a promising sustainable strategy to prevent the proliferation of above-ground fungal pathogens (Köberl et al., 2013; Bailly and Weisskopf, 2017; Garbeva and Weisskopf, 2020). Bruisson et al. have identified in grapevine leaf microbiome two *Bacillus subtilis* and *B. cereus* strains able to inhibit the growth of *Phytophthora infestans*, putatively through the emission of volatile compounds identified among pyrazines, chalconoids and tryptophan-derivatives.

The study by Teimoori-Boghsani et al. sheds light on another aspect of great interest, such as the potential of endophytes isolated from *Salvia abrotanoides* (Kar.) to induce synthesis of the bioactive diterpenoid cryptotanshinone by the plant and while doing that to produce the same molecule independent of the host. Their findings confirm the ability of endophytes to hijack the host’s metabolic setups while providing an interesting basis for agricultural and pharmaceutical exploitation of medicinal plants for the production of higher amounts of bioactive constituents.

## Combining Classical and Modern Approaches to Study the Plant-Associated Microbiota

In recent years, it has become clear that the complex structure of plant-associated microbial communities is a major driver of plant health (Lebeis et al., 2012; Bulgarelli et al., 2013), and a deeper understanding of the endophytic microbiome has the potential to become a pivotal tool for reducing the incidence of plant disease (Bloemberg and Lugtenberg, 2001).

In recent decades, plant microbial ecology has experienced a relentless proliferation of available techniques, and this multiplicity of approaches is also reflected in the studies gathered in our Research Topic. In exploring a wide array of different plant environments, the proposed studies ranged from culture-dependent screenings, where bacterial and fungal communities were profiled using PCR amplification of 16S ribosomal RNA gene or Internal transcribed spacer (ITS), as in Abdelshafy Mohamad et al., Teimoori-Boghsani et al., Bruisson et al., toward high-throughput technologies, as in the metagenomics strategy proposed by Liu et al., Araujo et al., Elsayed et al., and Anguita-Maeso et al.

This recourse to NGS technologies has provided a fundamental contribution, as they allow to reveal the presence of even rare microbial species and the interactions between these complex communities and their hosts, reaching a depth of resolution previously unimaginable (Bentley et al., 2008; Lebeis et al., 2012). Nevertheless, the importance of combining both culture-dependent and -independent methods to characterize the plants’ microbiota was nicely illustrated by Anguita-Maeso et al., and provided support to the notion that no one method can capture the plant microbiome in its entirety. This notion appears to be especially relevant to the characterization of endophytic communities inhabiting nutritionally poor environments, such as the xylem vessels in perennial crops (Aranda et al., 2011; Mendes et al., 2011; Dissanayake et al., 2018).

## The Rising Role of *Streptomyces* and Soil-Born Endophytes

As reviewed by Romano et al., the availability of the identification methods, not merely based on morphological characteristics, is of paramount importance to complement traditional methodologies in screening persistence of bioinoculants in the rhizosphere and their pattern of synergy with native microorganisms sharing the same niche. The interactions between rhizosphere and endosphere microbiomes and its dynamics have proved to play a critical role in shaping the agronomic traits of crop plants (Schlaeppi and Bulgarelli, 2015) and their study may help to identify effective biocontrol strains that can promote crop yield and reduce the consequences of serious infestations.

Among rhizosphere colonizers *Streptomyces spp*. are building a reputation as biocontrol agents against mycotoxigenic fungi, including numerous *Fusarium spp*. threatening cereal crops (Palazzini et al., 2007; Jung et al., 2013; Colombo et al., 2019). Colombo et al. identified an effective *Streptomyces* strain that was able to dramatically reduce Fusarium head blight symptoms in wheat and to prevent pathogen spread. Liu et al. provided evidence that *Streptomyces* strains isolated from *Glycine max* rhizosphere show excellent growth-promoting activity in soybean, in addition to delivering an important antagonistic activity against Sclerotinia stem rot disease. This two-side beneficial activity also resulted in the efforts of Araujo et al. who demonstrated the successful application of *Streptomyces* strains to accelerate the maturation of wheat heads and positively interfere with root microbiome challenged with severe *R. solani* infestation.

## Concluding Remarks and Future Challenges

Summarizing its overall heterogeneous composition, this Research Topic well represents the variety of experimental approaches and possible directions in studying this broad and very attractive area of science. The collection of results, presented in the papers, opened a unique knowledge window to the determinants and mechanisms that regulate the dual plant-endophyte interplay, and at the same time, to increased levels of complexity of the tri-partite interaction with phytopathogenic agents that cause severe diseases. The proposed approaches provide insights crucial for the development of new agro-biotechnological strategies for plant protection that will improve food security and environmental sustainability.

We also see our effort as an opportunity to feed the debate on several open questions that clearly emerged from the proposed research. For instance, we may point out the need to complement traditional microbiological approaches with next-generation -omics technologies, to capture as much diversity as possible (Singh, 2019). There is also the necessity to strengthen the newly discovered evidence by providing bio-analytical methods that allow tracking the persistence of bioinoculants and to understand their relationships with the autochthonous microbial communities, in the environments where they are released. Last but not the least, the alert on possible biosafety issues. So far, we just gained a partial knowledge on metabolic profiles of the majority of the components of plant-associated above-ground communities. However, we should be aware that the absence of phylogenetic relationships with human pathogens does not imply that endophytes do not present any risk for our health. Biosafety characteristics should be well addressed before proceeding with the environmental application (Keswani et al., 2019).

Based on these grounds, we believe that the multidisciplinary approach, proposed in our compendium, may result in the best strategy stimulating fast scientific progress on this challenging issue.

## Author Contributions

MM wrote the draft and submitted the final version. OB, KP, DH, and AO edited the manuscript and provided critical review. All authors contributed to the article and approved the submitted version.

## Funding

The participation of KP was partially funded by the General Secretariat for Research and Technology (GSRT) under the PRIMA Programme (INTOMED Project). AO was supported by the Faculty of Agriculture and Ministry of Education, Science and Technological Development, Republic of Serbia, contract no. 451-03-68/2020-14/200116.

## Conflict of Interest

The authors declare that the research was conducted in the absence of any commercial or financial relationships that could be construed as a potential conflict of interest.
